# Correction: design, synthesis and evaluation of cinnamic acid ester derivatives as mushroom tyrosinase inhibitors

**DOI:** 10.1039/c8md90024h

**Published:** 2018-05-10

**Authors:** Zhaojun Sheng, Siyuan Ge, Ximing Xu, Yan Zhang, Panpan Wu, Kun Zhang, Xuetao Xu, Chen Li, Denggao Zhao, Xiaowen Tang

**Affiliations:** a School of Chemical and Environmental Engineering , Wuyi University , Jiangmen 529020 , China . Email: wyuchemszj@126.com; b International Healthcare Innovation Institute (Jiangmen) , Jiangmen 529020 , China; c Institute of Bioinformatics and Medical Engineering , School of Electrical and Information Engineering , Jiangsu University of Technology , Changzhou 213001 , China

## Abstract

Correction for ‘Design, synthesis and evaluation of cinnamic acid ester derivatives as mushroom tyrosinase inhibitors’ by Zhaojun Sheng *et al.*, *Med. Chem. Commun.*, 2018, DOI: ; 10.1039/c8md00099a.



## 

The authors regret that the name for compound **5g** was incorrect in their manuscript (in the Abstract and Experimental section). The correct name for **5g** should be (*E*)-2-acetyl-5-methoxyphenyl 3-(4-fluorophenyl)acrylate.

The Royal Society of Chemistry apologises for these errors and any consequent inconvenience to authors and readers.

